# Screening for obstructive sleep apnea in elderly: performance of the Berlin and STOP-Bang questionnaires and the Epworth sleepiness scale using polysomnography as gold standard

**DOI:** 10.5935/1984-0063.20220033

**Published:** 2022

**Authors:** Paulo Henrique Godoy, Ana Paula Cassetta dos Santos Nucera, Andressa de Paiva Colcher, Jéssica Escorcio de Andrade, Davi da Silveira Barroso Alves

**Affiliations:** 1 Federal University of the State of Rio de Janeiro, Sleep Laboratory of the Federal University of the State of Rio de Janeiro - LABSONO UNIRIO - Rio de Janeiro - Brazil.; 2 Federal University of the State of Rio de Janeiro, Department of Quantitative Methods of the Federal University of the State of Rio de Janeiro - Rio de Janeiro - Brazil.

**Keywords:** Sleep Apnea, Obstructive, Mass Screening, Surveys and Questionnaires, Aged

## Abstract

**Objectives:**

Obstructive sleep apnea (OSA) affects approximately one third of the population and can reach 90% prevalence in the elderly. There are screening tools to track the disease, however, their performance may differ according to population characteristics. This study aims to determine sensitivity, specificity, predictive value, likelihood ratio, and accuracy of the Berlin (BQ) and STOP-Bang (S-Bang) questionnaires and the Epworth sleepiness scale (ESS), comparing their performances, using polysomnography (PSG) as a gold standard, in a sample of elderly.

**Material and Methods:**

The study was cross-sectional, retrospective, included patients aged 60 or older who underwent PSG type 1, regardless of the BQ, S-Bang and ESS results, during the period of June 1, 2017 to April 30, 2019. OSA diagnosis was by PSG in which the hypopnea apnea index was greater than or equal to 5.

**Results:**

Sixty-two patients were evaluated; the prevalence of OSA was 72.58%. The mean age in the sample with OSA was 73.0±8.4 years and without it was 74.7±8.1 years. The sample was predominantly female, 58.1% with OSA. The BQ showed the best results for specificity, predictive value, likelihood ratio and accuracy. S-Bang had the best result for sensitivity and ESS showed the worst results. The BQ odds ratio showed that an individual with a positive BQ has 335% more chance of developing OSA.

**Conclusion:**

The QB showed the best performance in the measures for identifying OSA, for a sample of elderly individuals, with a predominance of females and a high prevalence of the disease.

## INTRODUCTION

Obstructive sleep apnea (OSA) is the most common type of severe sleep-related breathing disorders^1,2^. Its overall prevalence is high, varying from 9% to 38% in the general population and increasing with age. In some groups of elderly people it reaches a rate of 90% prevalence in men and 78% in women^3,4^. Regarding the incidence of OSA, this is often underestimated, affecting between 2% and 5% of the middleaged population, however this percentage can change with aging^5^. There are studies that have estimated OSA incidence rates of 5.6% to 60% in people over 65 years of age and aging has been linked to an increase in the incidence of OSA^5^.

Due to the potentially serious adverse consequences associated with untreated OSA, prompt diagnosis and treatment are essential^6^. All-night polysomnography (PSG) is considered the gold standard exam for the diagnosis of OSA^2,7^. However, its use in the public health system is limited due to its cost and complexity^2,8,9^. A suitable screening method could be advantageous to detect those at higher risk, with follow-up and further evaluation using PSG^10^.

A variety of screening tools are employed for the evaluation of OSA. The Berlin (BQ) and STOP-Bang (S-Bang) questionnaires and the Epworth sleepiness scale (ESS) are the most frequently used^11^.

The BQ was the result of the Conference on Sleep in Primary Care, held in April 1996 in Berlin, Germany, a gathering that involved 120 primary care physicians from the United States of America (USA) and Germany^12^. Questions are divided into three categories. In category 1, there are four questions related to snoring and one about breathing pauses during sleep. In category 2, the questions refer to fatigue and tiredness, in addition to a question about sleep during the act of driving. Category 3 is related to body mass index (BMI) and the presence of systemic arterial hypertension (SAH). In the end, two or more positive categories can indicate high risk for OSA^13^. Despite being widely used in clinical practice, these tools have variations in sensitivity and specificity in different groups of patients, depending on age, gender and the presence of comorbidities^12,14,15^.

The STOP-Bang questionnaire was developed at the University of Toronto, Canada, initially for use in surgical patients and later for clinical patients^16^. It is an easy-to-execute method that is self-administered and consists of a series of eight questions referring to snoring, daytime fatigue, apnea, SAH, BMI, neck circumference, age and gender. The answers are yes and no, and the presence of at least 3 positive answers characterizes the individual at high risk for OSA^16^. Although widely used, there is still no consensus in the literature on subsequent indication of PSG based solely on S-Bang results^17^.

ESS was developed by Dr. Johns Murray, in 1991^18^, and was conceived based on observations related to the nature and occurrence of daytime sleepiness. The questionnaire is selfadministered, and individuals are asked to rate the probability of napping or falling asleep in eight different everyday situations on a scale of 0-3, generating a possibility of a result that varies from 0 to 24 points. Scores above 10 suggest the diagnosis of excessive daytime sleepiness (EDS). Although some authors highlight the subjectivity of this instrument^19^, Murray^18^ asserts its objectivity and points out that, like any other method, its use depends on understanding, interpretation and honesty in the patient’s responses^20^. It is a quick scale, easy to apply and does not involve any costs^21^.

However, studies show relevant variations in these tools’ sensitivity and specificity, depending on the characteristics of the individuals to whom such tools are applied, such as gender and age^22^.

The aim of this study is to verify sensitivity, specificity, positive and negative predictive value, and positive and negative likelihood ratio, and accuracy in the Berlin and STOP-Bang questionnaires, and the Epworth sleepiness scale using PSG as a gold standard, comparing the performance of these tools applied to a sample of elderly patients.

## MATERIAL AND METHODS

### Study design

The study is part of the project entitled “Obstructive sleep apnea syndrome in adult individuals: risk analysis using measurement tools in clinical practice and the association of risk factors and preexisting diseases”, approved by the ethics and research committee of the Gaffrée e Guinle University Hospital (*Hospital Universitário - Gaffrée e Guinle - HUGG*), through *Plataforma Brasil*, under number 3.298.539 in May 2019.

This is a cross-sectional, retrospective study, whose information came from elderly individuals, participants of the interdisciplinary program to promote health and quality of life for the elderly, the Renascer Group at HUGG. Participants were referred to the Sleep Laboratory of Federal University of the State of Rio de Janeiro (LABSONO UNIRIO).

### Materials

Demographic information about the individuals was collected using the LABSONO consultation data sheet and from medical records and the Renascer Group at HUGG. The data that make up the BQ and S-Bang tools, and the ESS, as well as the PSG results were collected using the LABSONO consultation data spreadsheet. These data refers to the period from June 1, 2017 to April 30, 2019.

The inclusion criteria were: all patients aged 60 years or over; the BQ and S-Bang questionnaires and ESS had to have been applied in all patients, they all had to have underwent PSG at HUGG, regardless of the results of the BQ, S-Bang and ESS, and the result was available in the LABSONO spreadsheet were included in the study.

Patients were excluded from the study if information and the results of the BQ, S-Bang, ESS and PSG were not available in the LABSONO spreadsheet or in the medical records of HUGG or the Renascer Group did not include the study analysis variables and those who underwent PSG, but already had a diagnosis of OSA and/or were in treatment.

All included patients underwent polysomnography performed in a sleep laboratory (PSG type 1)^23^, in the LABSONO. The OSA diagnosis was obtained via PSG type 1 in individuals whose apnea hypopnea index (AHI) was greater than or equal to 5.

The degree of apnea was classified according to the guidelines and recommendations for diagnosis and treatment of adult obstructive sleep apnea syndrome of the Brazilian Sleep Association^24^. Thus, patients with an AHI greater than or equal to 5 and less than or equal to 15 per hour of sleep were considered to have mild OSA. Those with an AHI greater than 15 and less than or equal to 30 per hour of sleep as moderate OSA and greater than 30 per hour of sleep as severe OSA.

### Statistical analysis

Statistical analysis was performed using the R program^25^ and results are presented as absolute numbers or frequencies, mean ± standard deviation, as appropriate. The T or Wilcoxon test was used to compare quantitative variables and the chisquare or Fisher’s exact test was used for qualitative variables.

Sensitivity (SEN), specificity (SPEC), positive (PPV) and negative (NPV) predictive value, and positive (LR+) and negative (LR-) likelihood ratios and accuracy (ACC) were estimated for each one of the three tools in relation to PSG, considered the gold standard.

There was also an investigation of the association of the tools with OSA in the sample through the chi-square test. The variables BQ, S-Bang and ESS were also applied in a univariate model and the odds ratio (OR) was estimated for each one.

## RESULTS

The sample consisted of 74 patients who were identified using the LABSONO data sheet, which contained patients from the RENASCER Group, from June 1, 2017 to April 30, 2019. Among these, 62 patients met the inclusion criteria. Of the 62 patients included, 45 had OSA, constituting 72.58% prevalence.

Regarding the characteristics of the patients who constituted the sample, the mean age was 73.5±8.3 years. For those with OSA it was 73.0±8.4 years and for those without OSA it was 74.7±8.1 years. Female participants were predominant in the sample, representing 58.1% of those with OSA and 24.2% without OSA. All patients in the sample declared themselves retired, reporting domestic activities. Patients with OSA had a higher body mass index (BMI) and there was an association in the exploratory data analysis performed. Considering preexisting diseases, systemic arterial hypertension (SAH) was the most frequent. It was present in more than 80% of the sample and in 86.7% in the group of patients with OSAS, but there was no association. Among the three analyzed tools, the Berlin questionnaire was the only one that showed an association in the exploratory analysis (Table 1).

**Table 1:** Characteristics of the patients with and without obstructive sleep apnea, diagnosed according to polysomnography, including the analyzed screening tools.

Variables	Sample (n=62)	With OSA (n=45)	Without OSA (n=17)	*p*-value
**Age** Mean SD	73.0±8.4	73.0±8.4	74.7±8.1	0.49
**Sex** Man	>11	9	>2	0.71
Woman	51	36	15	
**BMI** Mean SD	>28.40±6.4	29.2±6.6	>26.20±5.7	**0.03**
**SAH** Presence	>51	39	>12	0.15
Absence	11	5	5	
**Diabetes mellitus** Presence	>21	16	>5	0.65
Absence	41	29	12	
**Dyslipidemia** Presence	>32	23	>9	0.90
Absence	30	22	8	
**Chronic IHD** Presence	>17	10	>7	0.20
Absence	45	35	10	
**Asthma** Presence	>6	4	>2	0.66
Absence	56	41	15	
**Depression** Presence	>13	8	>5	0.32
Absence	49	37	12	
**BQ** Positive	>34	29	>5	**0.01**
Negative	28	16	12	
**S-Bang** Positive	>42	32	>10	0.35
Negative	20	13	7	
**ESS** Positive	>22	17	>5	0.54
Negative	40	28	12	

Abbreviations: OSA = Obstructive sleep apnea; BMI = Higher body mass index; SAH = Systemic arterial hypertension; Chronic IHD = Chronic ischemic heart disease; BQ = Berlin questionnaire; S-Bang = STOP-Bang questionnaire; ESS = Epworth sleepiness scale.

As for the grade of OSA, it was found that among the 45 patients, 17 had mild, 18 moderate and 10 had severe. Table 1 shows the characteristics of the patients with and without OSA, diagnosed using PSG, including the number of individuals identified with and without OSA, according to the screening tools applied in the sample.

As in the exploratory analysis, in the univariate modeling performed for the three tools, only the BQ showed an association with OSA. The odds ratio showed that an individual with a positive BQ has 335% more chance of developing OSA than an individual with a negative BQ (Table 2).

**Table 2:** Univariate modeling of screening tools for association with obstructive sleep apnea and odds ratio.

Tools	*p*-value	OR (95%CI)
BQ	0.02	4.35 (1.30-14.57)
S-Bang	0.36	1.72 (0.54-5.50)
ESS	0.54	1.46 (0.44-4.86)

Abbreviations: BQ = Berlin questionnaire; S-Bang = STOP-Bang questionnaire; ESS = Epworth sleepiness scale.

With regard to SEN, the S-Bang was the instrument with the best performance, of 71%. The BQ and the ESS performed better in identifying individuals without OSA, with a SPEC of 71% for both tools. The ACC was low for all tools, with the BQ showing the best results, but with little difference in relation to the S-Bang, 66% and 62%, respectively (Table 2).

If the S-Bang was the tool that most identified patients with OSA in the presence of the disease (SEN), the BQ was the one that, when it had a positive and negative result, presented the highest frequency of patients with and without OSA, PPV and NPV, respectively (Tables 1 and 2). The PPV above 75% for the three tools is notable, a fact expected due to the high prevalence of the disease in the sample.

The BQ stood out, among the tools, with the best results for the PPV, NPV, LR+ and LR- (Table 3).

**Table 3:** Analysis of screening tools for OSA according to sensitivity, specificity, positive and negative predictive value, positive and negative likelihood ratio, and accuracy, in the sample.

Measures	BQ	S-Bang	ESS
SEN	0.64 (0.49-0.78)	0.71 (0.56-0.84)	0.38 (0.24-0.53)
SPEC	0.71 (0.44-0.90)	0.41 (0.18-0.67)	0.71 (0.44-0.90)
PPV	0.85 (0.69-0.95)	0.76 (0.61-0.88)	0.77 (0.55-0.92)
NPV	0.43 (0.24-0.63)	0.35 (0.15-0.59)	0.30 (0.17-0.47)
LR+	2.19 (1.02-4.72)	1.21 (0.78-1.88)	1.28 (0.56-2.94)
LR-	0.50 (0.31-0.83)	0.70 (0.34-1.46)	0.88 (0.60-1.29)
ACC	0.66 (0.53-0.78)	0.62 (0.49-0.74)	0.47 (0.34-0.60)

Abbreviations: BQ = Berlin questionnaire; S-Bang = STOP-Bang questionnaire; ESS = Epworth sleepiness scale; SEN = Sensitivity; SPEC = Specificity; PPV = Positive predictive value; NPV = Negative predictive value; LR+ = Positive likelihood ratio; LR- = Negative likelihood ratio; ACC = Accuracy.

## DISCUSSION

The present study constitutes an important contribution to be observed in clinical practice as regards the application of screening tools for OSA, particularly the BQ, S-Bang and ESS, in a distinct and growing part of the Brazilian population, the elderly.

Regarding the results for the BQ, although high sensitivity (64%) - which is normally required for screening tests - was not found, the specificity was 71%, with consequent better PPV, 85%, and fewer false positive results. Since the sample had a high prevalence of OSA, it was expected that the PPV for all tools would be high. Among all the tools analyzed, the BQ was the one with the best PPV. The NPV was 43%, corroborating the low sensitivity.

The likelihood ratio (LR) combines sensitivity and specificity to estimate how much a given test contributes to the probability of disease detection, as compared to the prevalence of this disease^26^. In the sample, the BQ LR+ was 2.19, increasing the probability of OSA, since the higher the LR+, the greater the probability that a positive test result increases the probability of disease^26^. Among all tools, the BQ showed the best LR+, with a result greater than 1.6 times compared to the others. The LR- of the BQ, 0.5, also gave the best result among the analyzed tools, since the closer to zero, the lower the probability of illness in the presence of a negative test result.

The accuracy of the test takes into account the identification of individuals with a specific disease and the exclusion of those who do not have the disease^27^. In the present study, there was only one condition, OSA. The accuracy result for the tools was not good in any of them and as these were dichotomous tests, the sample size may have influenced this result. Among the tools, the BQ had the best accuracy (66%), closely followed by the S-Bang (62%), with the ESS delivering the worst result in terms of accuracy.

Still, in the exploratory analysis and univariate model, the BQ was the only instrument that showed an association with OSA, potentially corroborating the greater probability of identifying the disease in the sample through use of this particular screening instrument.

In the study conducted by Stelmach-Mardas et al. (2017)^28^ containing 64 patients with a mean age of 56.6 years, lower than that of the present study, using PSG as a diagnostic criterion, a high prevalence of OSA was also found: 72.58%, close to the prevalence in this investigation’s sample. It is possible that this shows that investigations in which PSG is used may find a higher prevalence of the disease, due to greater detection capacity. Although the studies cannot be compared, due to the different characteristics in the samples, it can be argued that despite the similar prevalence of disease, the SEN and SPEC, 87.2% and 11.8%, respectively, the PPV of 73.2% and 25% NPV, with an LR of approximately 1, described in the study by Stelmach-Mardas et al. (2017)^28^, were quite different from those found in the present investigation. The study by Miller et al. (2018)^29^ involving 170 people, with a mean age of 54.5 years and consisting of 51.76% males, showed that although the BQ was not the instrument with the best performance, it had a similar sensitivity (88.9%) to the Mardas study. Given these results, it is possible that the fact that the sample in this research was characterized by elderly individuals, may have contributed to better results in BQ measurements, compared to studies that considered gender and age without breaking them down by categories.

In 2016, the S-Bang was adapted and translated into Portuguese^30^ and, in 2017, it was validated for the identification of OSA in adults in Brazil^31^. In this study, the sample consisted of 456 adult patients, with a mean age of 43.7 years, 63.8% male. The method used for diagnosis was overnight PSG. High sensitivity of 83.5% was noted, in addition to low specificity of 45.5%. The accuracy for the sample was good, reaching 75.2%. Given these results, the author concluded that the S-Bang proved to be adequate for identifying OSA in the sample^31^.

In the present study, the S-Bang was the instrument with the best results for sensitivity, 71%, and the second best for accuracy, 62%, but with less specificity, 41%, the lowest among the three investigated tools. Higher sensitivity is important for screening tests, however, in the context of OSA, whose diagnosis includes a more costly test such as PSG, the most important question would be: once the individual is positive according to the instrument, what is the probability of the patient having the disease? Sensitivity does not answer this question, rather it shows the probability of a positive result, given that the patient has the disease^32^. The answer to the question lies in the predictive value, and in the present study both the PPV (76%) and the NPV (35%) were lower than those found for the BQ.

In interpreting the likelihood ratios, it is observed that the LR+ was greater than 1 (1.21), but it was the lowest value among the tools, therefore, showing a lesser probability that a positive test result increases the probability of disease detection when compared to the BQ. The LR- in the S-Bang questionnaire deviated more than zero than that observed in the BQ, meaning less possibility of identifying the lowest probability of disease in the presence of a negative result of the instrument when comparing them.

Most of the available studies investigating tools for sleep apnea screening were performed including individuals with a mean age between 50 and 60 years^33^. However, the investigation by Martins et al. (2020)^34^, which also involved the S-Bang, had a sample with similarities to the present study, mean age of 71 years, female predominance and high prevalence of OSA, 83%. The study showed high sensitivity and low specificity, PPV of 85% and NPV of 37%, with a LR+ of 1.237. Considering the proportions, these results were close to those found in the present investigation. The authors conclude that with a PPV of 85% in a sample with a prevalence of 83%, the risk of false positives, with an AHI cutoff point >5 events/h, is negligible and that, given a high prevalence of OSA in this age range, it may be wiser to indicate more objective tests as a first step in the investigation of OSA.

Regardless of the tools evaluated, what is exposed by Martins et al. (2020)^34^ in relation to a high prevalence for the disease could serve for the sample of this study. However, it is necessary to know the demographic characteristics and prevalence of diseases in a sample or population before drawing such a conclusion.

Another important aspect to be discussed in relation to the S-Bang questionnaire is that the risk markers present may have different characteristics in the young and old, which may require a restructuring of this tool^33^, and justify investigations in which the instrument is applied in different age and sex groups.

Excessive daytime sleepiness, identified in the ESS, despite not being related exclusively to OSA, has a significant correlation between the ESS scores and the AHI^35^. In 2009, Bertolazi et al.^35^ validated the use of ESS for Brazil, however, as the main objective was to develop the Portuguese version, and SEN, SPEC, VPP, VPN and SVR were not analyzed.

In the present study, the ESS showed good specificity, 71%, like the BQ. However, the sensitivity was very low, the worst among all tools, 38%. The high prevalence of OSA in the sample contributed to the higher PPV and the lower NPV value, as observed with the other tools. The LR+ and LR- were better, but close to those obtained for the S-Bang and worse when compared to the SVR of the BQ. The ESS was the instrument that presented the worst results of the measurements in the sample.

Exploratory analysis and univariate modeling corroborated these findings, as ESS had the worst result in terms of association with OSA, in addition to the lowest odds ratio.

In the study by Miller et al. (2018)^29^, which analyzed the ESS, among other tools, including 170 individuals, with a mean age of 54.5 years and a predominance of males, similar results were described, despite the different characteristics between the samples. Among the tools evaluated, ESS was the one with the highest specificity, 88.24%, and the worst sensitivity 17.92%, therefore seemingly the least desirable for screening for OSA_29_. Even in studies with larger samples, ESS is referred to as an inferior instrument for screening for the disease^36^. In the study conducted by Martins et al. (2020)^34^ mentioned above, which worked with a sample similar to the one in the present study, ESS also did not have good results, as it identified only 39% of individuals with the disease, a result very close to that found in the present study, 38%^33^. The authors also suggest that the assessment of sleepiness in the elderly may be less useful for tracking OSA than in adults of other age groups^34^.

It should be considered that although the evidence demonstrates that the isolated use of ESS is not ideal for screening for OSA, its combination with other tools, even using a lower cutoff point, can be useful when the objective is to increase the PPV of the instrument, as demonstrated in the study by Senaratna et al. (2019)^37^.

With regard to the characteristics of the individuals who constituted the sample, it is important to mention the high frequency of women found in the sample and the nonassociation of OSA with previous diseases in the sample. This fact may be related to the sample size, but on the other hand, it is a characteristic of the health promotion program for the elderly, which reflects the greater demand of women in the health system and the particularity of the characteristics of individuals assisted in programs such as this one. Even the mean BMI found in the sample refers to overweight, if we consider the largest standard deviation, there is at most grade 1 obesity. The difference in BMI between the group with and without OSAS was not large, but it was greater in the group with OSA. Also as in the investigation by Sforza et al. (2011)^38^, there was an association of BMI with the presence of OSA in the exploratory analysis, however, this was not confirmed in the univariate model. Perhaps the constitution of the elderly, with factors such as decreased muscle mass, greater adipose tissue and reduced height due to arching of the spine, can explain conflicting aspects of studies with the elderly that involve these measures.

Finally, it is important to mention that the limitation of the present study was the small sample size. However, it should be considered that this is a retrospective study, whose sample of patients came from a health promotion program for the elderly and not from a specific outpatient clinic for the investigation of sleep disorders. It emphasizes the inclusion criteria in the study, in which elderly patients should have completed all the questionnaires involved in the investigation and performed the PSG in a sleep laboratory, type 1.

## CONCLUSION

Despite the small sample of patients, the study suggests the importance of screening elderly individuals for OAS, even if they come from a prevention or health promotion program, and shows the performance of the instruments most commonly used for this type of screening, in these individuals, although future investigations will be carried out with a larger sample.

In conclusion, among the tools evaluated, the BQ showed the best results in measures, specificity, predictive values and LR in identifying OSA in a sample of individuals over 60 years of age, with a predominance of females and a high prevalence of the disease. The STOP-Bang questionnaire showed intermediate performance and ESS the worst results.

## References

[1] Thurnheer R (2011). Diagnostic approach to sleep-disordered breathing. Expert Rev Respir Med.

[2] Abbasi A, Gupta SS, Sabharwall N, Meghrajanil V, Sharma SS, Kamholzl S (2021). A comprehensive review of obstructive sleep apnea. Sleep Sci.

[3] Senaratna CV, Perret JL, Lodge CJ, Lowe AJ, Campbell BE, Matheson MC (2016). Prevalence of obstructive sleep apnea in the general population: a systematic review. Sleep Med Rev.

[4] Tufik S, Santos-Silva R, Taddei JA, Bittencourt LRA (2010). Obstructive sleep apnea syndrome in the Sao Paulo epidemiologic sleep study. Sleep Med.

[5] Iannella G, Magliulo G, Lo Iacono C, Bianchi G, Polimeni A, Greco A (2020). Positional obstructive sleep apnea syndrome in elderly patients. Int J Environ Res Public Health.

[6] Chung F, Abdullah HR, Liao P (2016). STOP-Bang questionnaire: a practical approach to screen for obstructive sleep apnea. Chest.

[7] Patil SP, Schneider H, Schwartz AR, Smith PL (2007). Adult obstructive sleep apnea: pathophysiology and diagnosis. Chest.

[8] Moura WL, Moura CS, Silva TSO, Sipaúba GMO, Moura MSL, Martins GAS (2018). Prevalência do risco da Sindrome da Apneia Obstrutiva do Sono na população adulta de uma capital brasileira. Rev Fac Odontol.

[9] Viegas CAA (2010). Epidemiology of sleep-disordered breathing. J Bras Pneumol.

[10] Senaratna CV, Perret JL, Matheson MC, Lodge CJ, Lowe AJ, Cassim R (2017). Validity of the Berlin questionnaire in detecting obstructive sleep apnea: a systematic review and meta-a nalysis. Sleep Med Rev.

[11] Shahid A, Wilkinson K, Marcu S, Shapiro CM, Shahid A, Wilkinson K, Marcu S, Shapiro CM (2012). STOP, THAT and one hundred other sleep scales.

[12] Netzer NC, Stoohs RA, Netzer CM, Clark K, Strohl KP (1999). Using the Berlin questionnaire to identify patients at risk for the sleep apnea syndrome. Ann Intern Med.

[13] Zancanella E, Haddad FM, Oliveira LAMP, Nakasato A, Duarte BB, Soares CFP (2014). Apneia obstrutiva do sono e ronco primário: diagnóstico. Braz J Otorhinolaryngol.

[14] Araújo-Melo MH, Neves DD, Ferreira LVMV, Moreira MLV, Nigri R, Simões SMG (2016). Questionários e escalas úteis na pesquisa da síndrome da apneia obstrutiva do sono. Rev Hosp Univ Pedro Ernesto.

[15] Vaz AP, Drummond M, Mota PC, Severo M, Almeida J, Winck JC (2011). Tradução do questionário de Berlim para língua portuguesa e sua aplicação na identificação da SAOS numa consulta de patologia respiratória do sono. Rev Port Pneumol.

[16] Chung F, Yegneswaran B, Liao P, Chung SA, Vairavanathan S, Islam S (2008). STOP questionnaire: a tool to screen patients for obstructive sleep apnea. Anesthesiology.

[17] Toronto Western Hospital (2012). The Official STOP-Bang questionnaire website [Internet].

[18] Johns MW. (1991). A new method for measuring daytime sleepiness: the Epworth sleepiness scale. Sleep.

[19] Guimarães C, Martins MV, Vaz Rodrigues L, Teixeira F, Santos J (2012). Escala de sonolência de Epworth na síndrome de apneia obstrutiva do sono: uma subjetividade subestimada. Rev Port Pneumol.

[20] Johns MW (2000). Sensitivity and specificity of the multiple sleep latency test (MSLT), the maintenance of wakefulness test and the Epworth sleepiness scale: failure of the MSLT as a gold standard. J Sleep Res.

[21] Nogueira C, Azevedo IO, Bentes P, Magalhães A, Lacerda CA, Vera M (2013). The effectiveness of the epworth sleepiness scale as an auxiliary resource in the diagnosis of obstructive sleep apnea syndrome. Rev Bras Promoç Saúde.

[22] Musman S, Passos VMA, Silva IBR, Barreto SM (2011). Avaliação de um modelo de predição para apneia do sono em pacientes submetidos a polissonografia. J Bras Pneumol.

[23] Kuna ST, Badr MS, Kimoff RJ, Kushida C, Lee-Chiong T, Levy P (2011). An official ATS/AASM/ACCP/ERS workshop report: Research priorities in ambulatory management of adults with obstructive sleep apnea. Proc Am Thorac Soc.

[24] Haddad F, Bittencourt L (2013). Recomendações para o diagnóstico e tratamento da síndrome da apneia obstrutiva do sono no adulto.

[25] R Core Team (2008). R: A language and environment for statistical computing [Internet].

[26] Ferreira JC, Patino CM (2018). Understanding diagnostic tests. Part 3. J Bras Pneumol.

[27] Oliveira GM, Camargo FT, Gonçalves EC, Duarte CVN, Guimarães CA (2010). Systematic review of diagnostic tests accuracy: a narrative review. Rev Col Bras Cir.

[28] Stelmach-Mardas M, Iqbal K, Mardas M, Kostrzewska M, Piorunek T (2017). Clinical utility of Berlin questionnaire in comparison to polysomnography in patients with obstructive sleep apnea. Adv Exp Med Biol.

[29] Miller JN, Kupzyk KA, Zimmerman L, Pozehl B, Schulz P, Romberger D (2018). Comparisons of measures used to screen for obstructive sleep apnea in patients referred to a sleep clinic. Sleep Med.

[30] Fonseca LBM, Silveira EA, Lima NM, Rabahi MF (2016). STOP-Bang questionnaire: translation to Portuguese and cross-cultural adaptation for use in Brazil. J Bras Pneumol.

[31] Duarte RLM, Fonseca LBM, Silveira FJM, Silveira EA, Rabahi MF (2017). Validação do questionário STOP-Bang para a identificação de apneia obstrutiva do sono em adultos no Brasil. J Bras Pneumol.

[32] Ferreira JC, Patino CM (2017). Understanding diagnostic tests. Part 1. J Bras Pneumol.

[33] Chiu HY, Chen PY, Chuang LP, Chen NH, Tu YK, Hsieh YJ (2017). Diagnostic accuracy of the Berlin questionnaire, STOP-BANG, STOP, and Epworth sleepiness scale in detecting obstructive sleep apnea: a bivariate meta-analysis. Sleep Med Rev.

[34] Martins EF, Martinez D, Cortes AL, Nascimento N, Brendler J (2020). Exploring the STOP-BANG questionnaire for obstructive sleep apnea screening in seniors. J Clin Sleep Med.

[35] Bertolazi AN, Fagondes SC, Hoff LS, Pedro VD, Barreto SSM, Johns MW (2009). Portuguese-language version of the Epworth sleepiness scale: validation for use in Brazil. J Bras Pneumol.

[36] Penafiel FS, Poniachik JG, López AC, Monasterio JU, Patino OD (2018). Accuracy of sleep questionnaires for obstructive sleep apnea syndrome screening. Rev Med Chil.

[37] Senaratna CV, Perret JL, Lowe A, Bowatte G, Abramson MJ, Thompson B (2019). Detecting sleep apnea syndrome in primary care with screening questionnaires and the Epworth sleepiness scale. Med J Aust.

[38] Sforza E, Chouchou F, Collet P, Pichot V, Barthélémy JC, Roche F (2011). Sex differences in obstructive sleep apnoea in an elderly French population. Eur Respir J.

